# Poly(ADP-ribose) potentiates ZAP antiviral activity

**DOI:** 10.1371/journal.ppat.1009202

**Published:** 2022-02-07

**Authors:** Guangai Xue, Klaudia Braczyk, Daniel Gonçalves-Carneiro, Daria M. Dawidziak, Katarzyna Sanchez, Heley Ong, Yueping Wan, Kaneil K. Zadrozny, Barbie K. Ganser-Pornillos, Paul D. Bieniasz, Owen Pornillos

**Affiliations:** 1 Department of Molecular Physiology and Biological Physics, University of Virginia, Charlottesville, Virginia, United States of America; 2 Laboratory of Retrovirology, Howard Hughes Medical Institute, The Rockefeller University, New York, New York, United States of America; Fred Hutchinson Cancer Research Center, UNITED STATES

## Abstract

Zinc-finger antiviral protein (ZAP), also known as poly(ADP-ribose) polymerase 13 (PARP13), is an antiviral factor that selectively targets viral RNA for degradation. ZAP is active against both DNA and RNA viruses, including important human pathogens such as hepatitis B virus and type 1 human immunodeficiency virus (HIV-1). ZAP selectively binds CpG dinucleotides through its N-terminal RNA-binding domain, which consists of four zinc fingers. ZAP also contains a central region that consists of a fifth zinc finger and two WWE domains. Through structural and biochemical studies, we found that the fifth zinc finger and tandem WWEs of ZAP combine into a single integrated domain that binds to poly(ADP-ribose) (PAR), a cellular polynucleotide. PAR binding is mediated by the second WWE module of ZAP and likely involves specific recognition of an adenosine diphosphate-containing unit of PAR. Mutation of the PAR binding site in ZAP abrogates the interaction *in vitro* and diminishes ZAP activity against a CpG-rich HIV-1 reporter virus and murine leukemia virus. In cells, PAR facilitates formation of non-membranous sub-cellular compartments such as DNA repair foci, spindle poles and cytosolic RNA stress granules. Our results suggest that ZAP-mediated viral mRNA degradation is facilitated by PAR, and provides a biophysical rationale for the reported association of ZAP with RNA stress granules.

## Author summary

Zinc-finger antiviral protein (ZAP), also known as poly(ADP-ribose) polymerase 13 (PARP13), functions as a host defense mechanism against viruses, including important human pathogens such as hepatitis B virus and type 1 human immunodeficiency virus (HIV-1). ZAP recognizes and binds viral RNA by virtue of their nucleotide composition and directs selective degradation of these viral RNA. Here, we report the X-ray crystal structures of ZAP’s central domain, which we found to bind poly(ADP-ribose) (PAR), a cellular polynucleotide. In cells, PAR is associated with macromolecular assemblages that are implicated in virus inhibition and antiviral signaling. We confirm through biochemical experiments that ZAP indeed binds PAR, both in vitro and in cells. However, the PAR-binding activity of ZAP is not essential to its antiviral function. Instead, we find that PAR binding is an ancillary activity that contributes to the potency of ZAP-mediated virus inhibition.

## Introduction

Cells encode a variety of nucleic acid sensors that detect the presence of viral RNA or DNA by virtue of non-self features or inappropriate localization. The zinc-finger antiviral protein ZAP (also known as poly(ADP-ribose) polymerase 13 or PARP13) is one such sensor and selectively binds to viral messenger RNA or viral RNA genomes [1,2]. The ZAP-bound RNA molecules are subjected to degradation or translational inhibition, which consequently decreases production of viral proteins and suppresses virus replication [1,3,4]. Depending on the virus, the action of ZAP can selectively suppress viral protein expression by up to 30-fold, while cellular protein expression levels remain largely unaffected [1].

**Fig 1:**
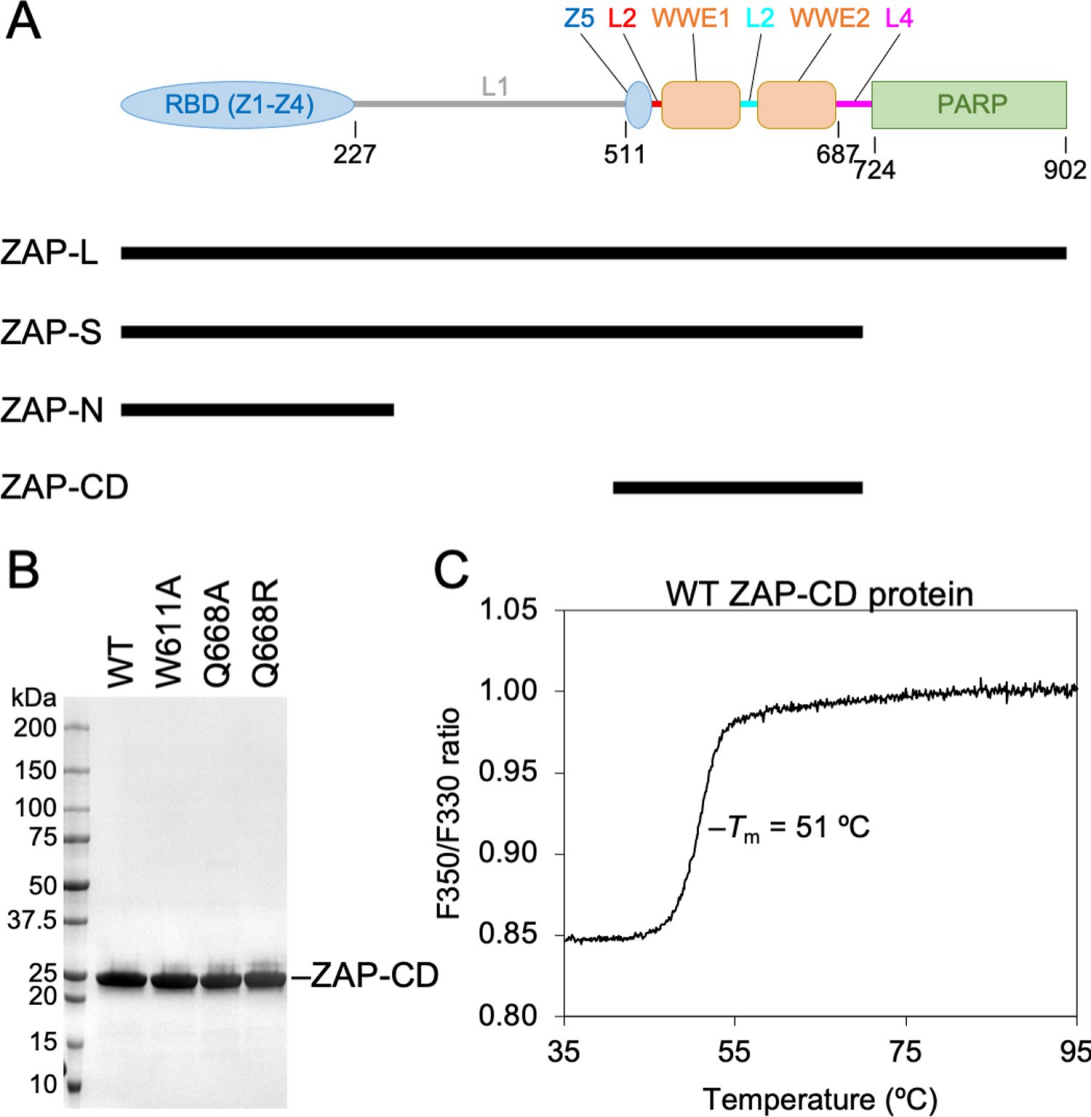
Modular domain organization of ZAP. (A) Domain diagram of the ZAP primary sequence. Modules are colored according to their structural properties (zinc fingers Z1, Z2, Z3, Z4 and Z5 in blue; WWE1 and WWE2 domains in orange; PARP in green; inter-domain linkers L1, L2, L3, and L4 in gray, red, cyan and magenta). Indicated below are the two major naturally-occurring splice isoforms (ZAP-L and ZAP-S), the minimally active antiviral fragment (ZAP-N), and the central domain described in this study (ZAP-CD). (B) SDS-PAGE profiles of purified recombinant ZAP-CD proteins used in this study. (C) Differential scanning fluorimetry profile of wild type (WT) ZAP-CD shows a single transition. The apparent melting temperature (*T*_m_) is 50.9 ± 0.1°C, determined with five independent protein preparations.

ZAP has a modular organization and is expressed as two major isoforms called ZAP-L and ZAP-S, that arise from alternative splicing and are distinguished by the presence of a C-terminal PARP or poly(ADP-ribose) polymerase-like domain (Fig 1A). Both isoforms contain an N-terminal RNA-binding domain (RBD) with four zinc fingers (here termed Z1 to Z4) that bind to CpG dinucleotides in RNA [5,6]. Vertebrate genomes are depleted of CpG content, and it is the relative scarcity of this dinucleotide in cellular RNA compared to susceptible viral RNA that explains selective ZAP-mediated degradation [7]. A truncated ZAP fragment (here called ZAP-N; Fig 1A) that essentially consists of only the RBD is both necessary and sufficient for directing viral RNA degradation [1]. However, there are indications that other ZAP domains are also important for its antiviral function. For example, the PARP-like domain in ZAP-L shows signatures of positive selection, as is found in a host protein that is locked in an antagonistic “arms race” with viruses [8]. The absence of the PARP-like domain and mutagenesis of its vestigial catalytic site are also reported to negatively affect ZAP antiviral activity [8,9].

In both ZAP-L and ZAP-S, the RBD is connected by a long linker segment to a fifth zinc finger (Z5) and two WWE domains (WWE1 and WWE2) (Fig 1A). These additional ZAP domains have unknown function, but WWE domains in other proteins are reported to have a general role in binding to poly(ADP-ribose) (PAR) [10,11]. PAR is a cellular polynucleotide that has been shown to function as a scaffold or collective docking site for multiple protein partners, thereby allowing for sustained co-localization of the components of cellular pathways [12,13]. Here, we show that Z5, WWE1 and WWE2 are sub-domains or modules that integrate into a composite fold, which we term the ZAP central domain (ZAP-CD). Structural and biochemical analyses revealed that ZAP-CD binds to PAR through the second WWE module. Both ZAP [14–18] and PAR [14,19] have been previously reported to localize to so-called RNA stress granules, which constitute a type of non-membranous cytoplasmic compartment that facilitates RNA turnover and antiviral responses [20,21]. Our studies suggest that PAR may coordinate the stable association of ZAP and its co-factors and thereby facilitate efficient recognition and/or degradation of ZAP-bound RNA.

## Results

### ZAP Z5, WWE1 and WWE2 form a single composite domain

**Fig 2:**
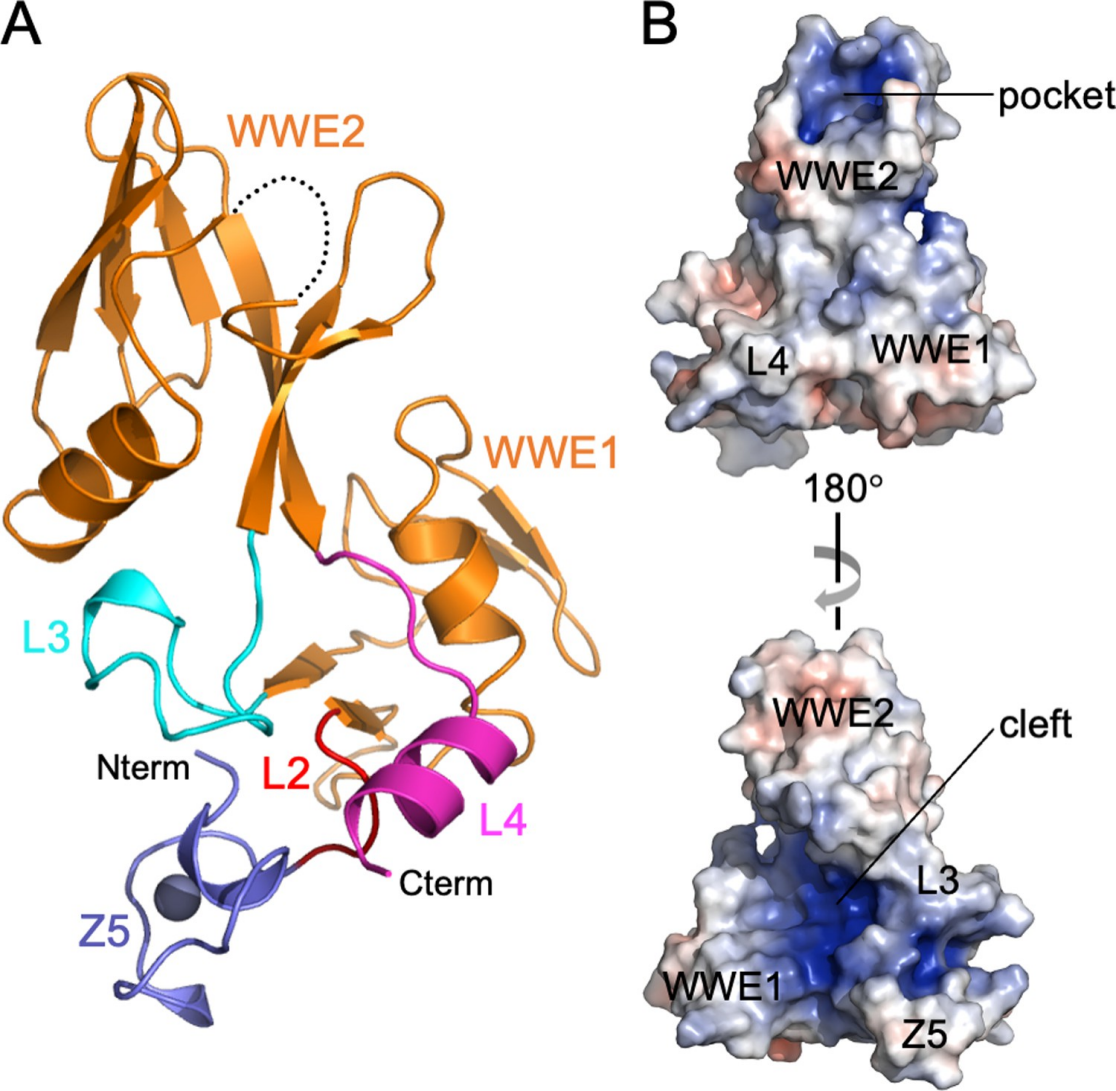
Structure of ZAP-CD. (A) Ribbons representation. Modules are colored according to Fig 1A (Z5 in blue, WWE1 and WWE2 in orange), as are the linkers (L2 in red, L3 in cyan, and L4 in magenta). The amino and carboxyl termini are also indicated. Black dashes denote a flexible loop in the second WWE module with very weak density and left unmodeled. (B) Orthogonal surface views colored according to electrostatic potential from red (negative) to blue (positive). The Z5, WWE1 and WWE2 modules are indicated, as are the putative isoADPr pocket (top) and the extended electropositive cleft (bottom).

We first aimed to define a protein construct from the central regions of ZAP that could be characterized biochemically. The Z5, WWE1 and WWE2 modules were insoluble when individually overexpressed recombinantly in *E*. *coli*, but a ZAP construct spanning residues 498–699 and containing all three was highly soluble and could be purified to homogeneity (Fig 1B). The purified ZAP-CD protein exhibited a single folding transition in thermal melting experiments (Fig 1C). We then determined the crystal structure of ZAP-CD to 2.5 Å resolution (*R*_work_/*R*_free_ = 0.22/0.26) (Fig 2A and Table 1). The Z5, WWE1 and WWE2 modules each form a compact fold or sub-domain. The core fold of the Z5 module is similar to the other four zinc fingers, Z1, Z2, Z3 and Z4, that comprise the RBD [22] (S1A and S1B Fig). The zinc modules differ primarily in loop sizes and configurations, with Z5 being most similar to Z4. Comparison with Z2, the principal CpG-binding module [5,6], revealed no correspondence in Z5 with the Z2 residues that contact RNA (S1C Fig). In ZAP-CD, close packing between the Z5, WW1 and WWE2 modules is mediated by well-ordered “linker” residues, which we term L2, L3 and L4 (colored in red, cyan and magenta in Fig 2A); although separated in sequence these linkers come together in the middle of the structure and glue together the three sub-domains. Thus, the three modules or sub-domains of ZAP-CD are integrated into a composite fold that likely behaves as a single functional unit.

**Table 1:** Crystallographic statistics.

	SeMet ZAP-CD	ZAP-CD + ADPr
**Diffraction Data**		
Beamline	APS 22ID	APS 22BM
Wavelength (Å)	0.97856	1.000
Processing program	HKL2000	HKL2000
Space group	P321	P321
Cell dimensions	*a* = *b* = 89.637 Å, *c* = 53.055 Å	*a* = *b* = 89.958 Å, *c* = 52.807 Å
	α = β = 90°, γ = 120°	α = β = 90°, γ = 120°
Resolution range, Å	50–2.50 (2.54–2.50)	50–1.97 (2.04–1.97)
*R*_sym_ / *R*_meas_ / *R*_pim_	0.14 (1) / 0.18 (1) / 0.03 (0.24)	0.11 (1) / 0.11 (1) / 0.03 (0.59)
CC1/2	0.998 (0.893)	0.987 (0.458)
Mean I/σ<I>	24.2 (5.0)	25.2 (1.1)
Completeness, %	99.5 (100)	100 (99.9)
Average redundancy	48.0 (24.1)	21.2 (13.3)
Wilson B-factor, Å^2^	35.6	24.1
**Phasing**		
Phasing program	PHENIX (phenix.autosol)	MOLREP
Method	SAD	Molecular replacement
No. of Se sites	5	n.a.
Figure of merit	0.38	n.a.
**Refinement Statistics**		
Refinement program	PHENIX (phenix.refine)	PHENIX (phenix.refine)
Resolution range	39–2.50 (2.70–2.50)	44–1.98 (2.10–1.98)
No. of unique reflections	8,296 (1,701)	15,156 (1218)
Reflections in free set	428 (89)	843 (82)
*R*_work_ / *R*_free_	0.22 (0.26) / 0.26 (0.29)	0.24 (0.29) / 0.27 (0.32)
No. of nonhydrogen atoms		
protein	1,447	1,446
zinc	1	1
ADPr	0	36
water	33	75
Average B-factor (Å^2^)		
protein	42.1	33.6
zinc	43.2	35.6
ADPr	0	52.8
water	34.7	29.8
Coordinate deviations		
bond lengths, Å	0.003	0.014
bond angles, °	0.530	1.039
**Validation and Deposition**		
Ramachandran plot		
favored, %	97.8	96.6
outliers, %	0	0
MolProbity clashscore	0.71	3.47
PDB ID	7KZH	7TGQ

Values in parenthesis are for the highest resolution shell.

Examination of the surface features and electrostatic potential of ZAP-CD revealed two major regions of interest: a deep pocket in the second WWE module (Fig 2B, top), which appeared suitable for binding an aromatic ligand (discussed in more detail below), and a deep cleft running along one side of the composite domain (Fig 2B, bottom). A similar cleft was observed in the tandem WWE fold of Deltex, which was proposed to be a polypeptide binding site [23]. In ZAP-CD, the cleft is extended by the Z5 module, which contributes positively-charged sidechains to the cleft walls (Fig 2B, bottom). The highly electropositive nature of the cleft suggests that it may also be well suited to bind negatively-charged, non-proteinaceous polymers such as polynucleotides.

### ZAP-CD contains a putative PAR binding site

**Fig 3:**
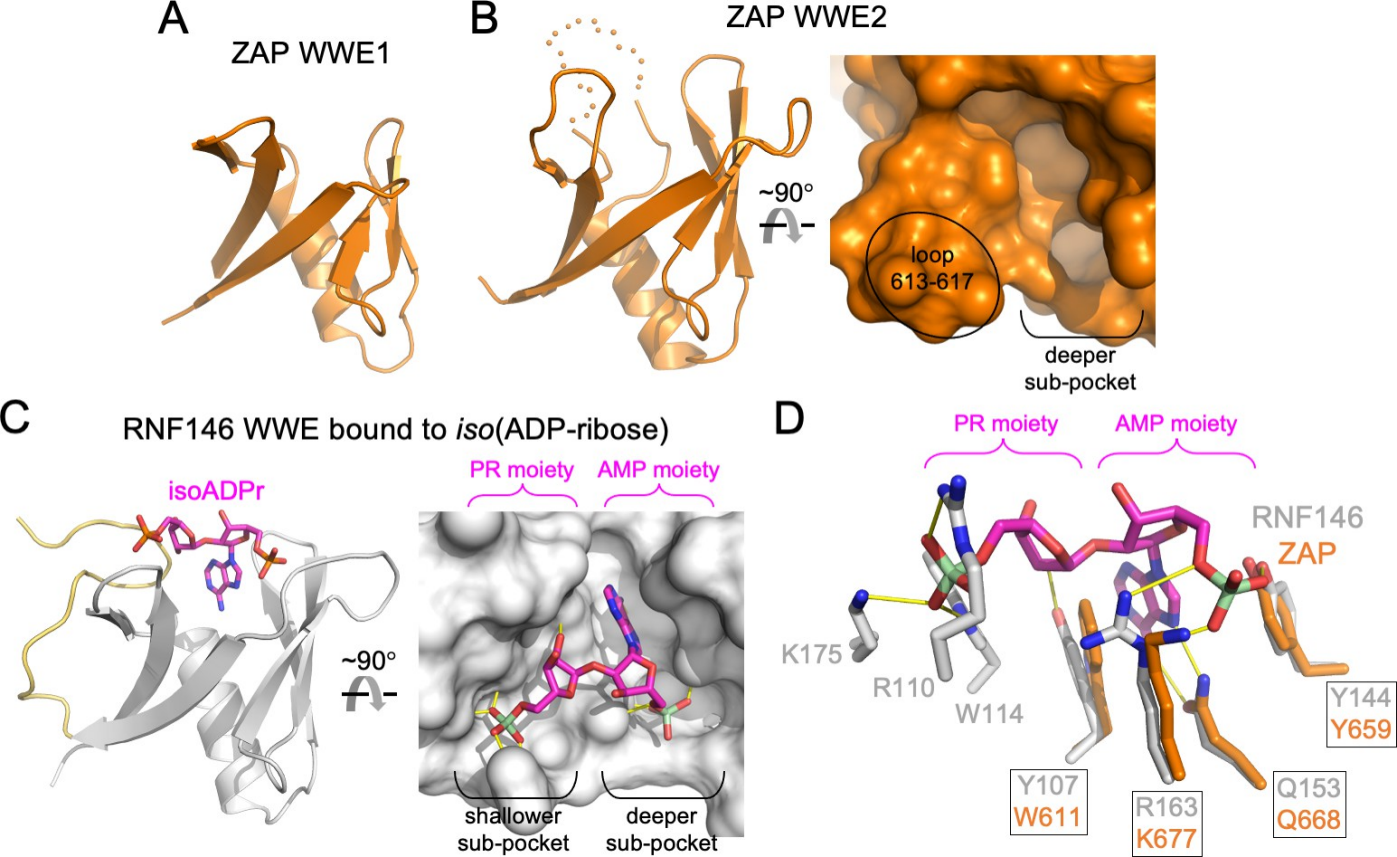
Structural analysis of the ZAP WWE modules. (A) Structure of ZAP WWE1. (B) Structure of ZAP WWE2 in ribbons (left) and surface representation (right). Relevant features are labeled. (C) Structure of RNF146 WWE with bound isoADPr ligand (PDB 3V3L) [11]. Left shows the protein in ribbons (gray with the C-terminal extension in yellow) and the ligand in sticks (magenta). Right shows the protein in surface representation. Relevant features are labeled. (D) View of pocket residues in the same orientation as the ribbons in B and C, after backbone-guided superposition of ZAP WWE2 (orange) and RNF146 WWE (gray) with bound isoADPr (magenta). Residues with structural equivalents in ZAP and RNF146 are boxed; residues with no equivalents are unboxed. Hydrogen bonds are shown in yellow.

The WWE domain was first described as an independent protein-folding module with a characteristic signature of conserved tryptophan, glutamate and arginine residues, and is found in many proteins that function in ubiquitination and PARylation pathways [10,11]. In ZAP, the two WWE modules share the same canonical β-strand/α-helix fold as expected, but differ considerably with regards to the loops connecting the β-strands (compare Fig 3A and Fig 3B). In the second WWE module, the loops are more extended and generate the abovementioned pocket (Fig 3B). Comparison with other known WWE structures revealed that a similar pocket is present in RNF146 (an E3 ubiquitin ligase involved in DNA repair) [11,24] (Fig 3C). In RNF146, the pocket is a nM-affinity binding site for *iso*(ADP-ribose) (isoADPr), which is a recognition unit of PAR. IsoADPr consists of adenosine monophosphate (AMP) and phosphoribose (PR) moieties connected by a glycosidic bond, and these two moieties occupy two sub-pockets in the RNF146 binding site (Fig 3C and 3D): (1) the AMP moiety buries its adenine ring in a deeper sub-pocket, making hydrogen bonds with a buried glutamine (Q153) and packing against an aromatic sidechain (Y107). Also, the phosphate group of the AMP moiety makes hydrogen bonds with an arginine (R163) and a tyrosine (Y144); (2) the PR moiety occupies a shallower sub-pocket, where a ribose hydroxyl and all the phosphate oxygen atoms are engaged in hydrogen bonds with four RNF146 sidechains.

Interestingly, whereas the deeper sub-pocket is conserved in ZAP, the shallower sub-pocket is not (Fig 3B). ZAP has structural equivalents to all the RNF146 residues that contact the AMP moiety (Fig 3D, boxed labels), but not the RNF146 residues that contact the PR moiety (Fig 3D, not boxed). Notably, RNF146 Y107, which demarcates the sub-pockets and interacts with both the AMP and PR moieties, is a tryptophan (W611) in ZAP (Fig 3D). This ZAP residue can therefore recapitulate stacking with the AMP adenine ring but not hydrogen bonding with the PR ribose. Furthermore, the shallower sub-pocket in ZAP is partly occluded by a loop (residues 613–617; indicated in Fig 3B). ZAP is also missing a C-terminal extension that in RNF146 loops back from the bottom of the domain to make up one side of its shallower PR sub-pocket (yellow in Fig 3C). One interpretation of these observations is that ZAP and RNF146 recognize distinct units of PAR; these distinct units would share a common AMP moiety. Alternatively, ZAP may also bind the isoADPr unit, but this would require remodeling of the binding site to generate the shallower sub-pocket or a different bound configuration of the PR moiety.

Consistent with our interpretation that ZAP can bind an AMP moiety of PAR, closer examination of our electron density maps within the deeper sub-pocket revealed residual unbiased difference densities, whose shapes are consistent with a bound adenine ring and a phosphate (S2A and S2B Fig). We surmise that some abundant small molecule containing an adenine ring and phosphate groups (e.g., ATP, ADP-ribose or similar) co-purified and co-crystallized with our recombinant ZAP-CD protein (see also Materials and Methods). Protein backbone-guided superposition of the ZAP-CD and RNF146 structures places the AMP moiety of the RNF146-bound isoADPr within these densities (S2C Fig).

### Structure of ZAP-CD in complex with ADP-ribose

**Fig 4:**
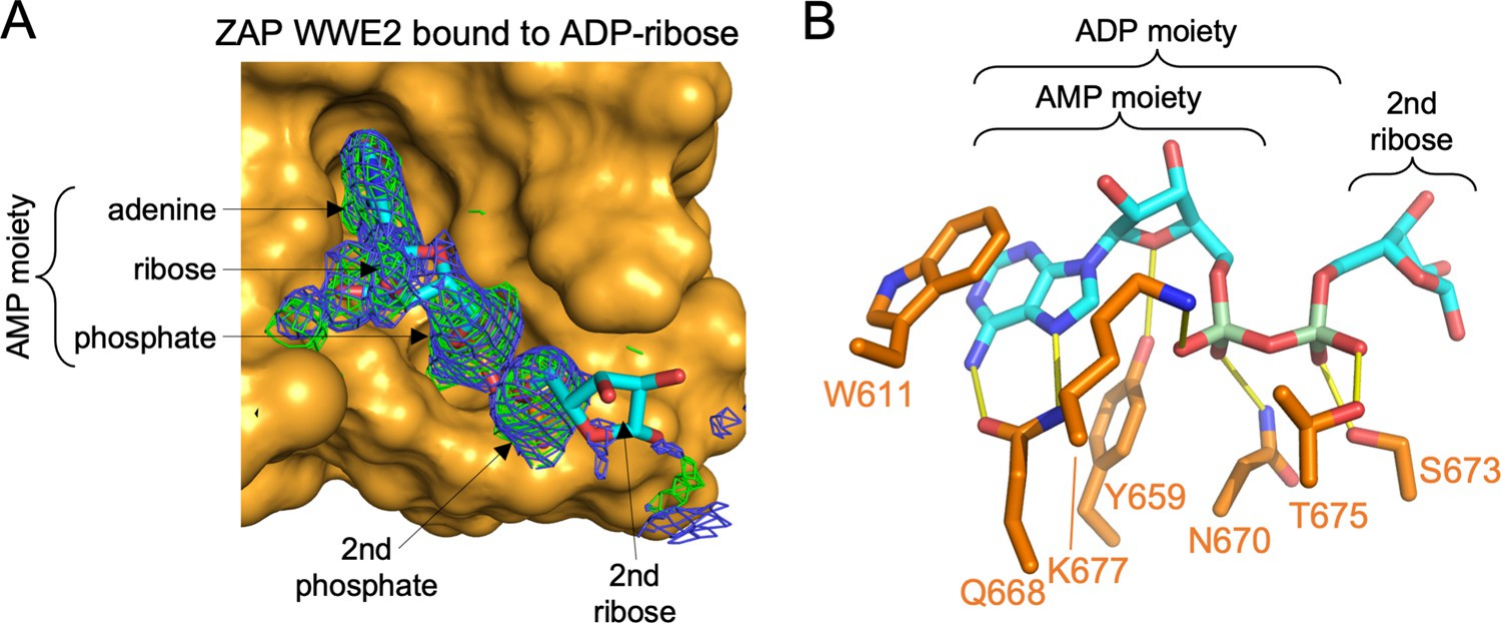
Structure of ZAP-CD in complex with ADPr. (A) Surface representation (orange) of the ZAP WWE2 pocket with the bound ADPr in stick representation (cyan), in the same orientation as Fig 3B, right panel. Blue mesh shows the 2mFo-DFc map for the ligand after refinement, contoured at 1σ. Green mesh shows the unbiased positive mFo-DFc densities prior to placement of ADPr, contoured at 2.5σ. (B) Details of the binding interaction, with ZAP residues shown in orange and the ADPr carbon atoms in cyan. Phosphate atoms are in pale green. Hydrogen bonds are in yellow.

To confirm our interpretation that ZAP binds to an AMP moiety, we crystallized ZAP-CD in the presence of excess ADP-ribose (ADPr), the repeating unit of PAR. The structure of ZAP-CD in complex with ADPr was refined against data extending to 2.0 Å resolution (*R*_work_/*R*_free_ = 0.24/0.27) (Fig 4 and Table 1). The protein conformation was largely unchanged. Consistent with the above analysis, densities for the entire AMP moiety were very well defined, and we found contacts to the adenine ring and first phosphate as predicted by the comparison with RNF146 (Fig 4A and 4B). The structure also revealed additional ZAP contacts with the AMP moiety, including a hydrogen bond between N670 and the first phosphate group (Fig 4B). More importantly, the structure further revealed strong density for the second phosphate (Fig 4A), which acts as hydrogen bond acceptor for S673 and T675 (Fig 4B). Density for the second ribose in ADPr was weak (Fig 4A), suggesting that this moiety does not significantly contribute to the interaction. Thus, our structural data suggest that the ZAP WWE2 pocket is configured to bind an adenosine diphosphate (ADP) moiety of PAR.

### ZAP-CD binds PAR *in vitro*

**Fig 5:**
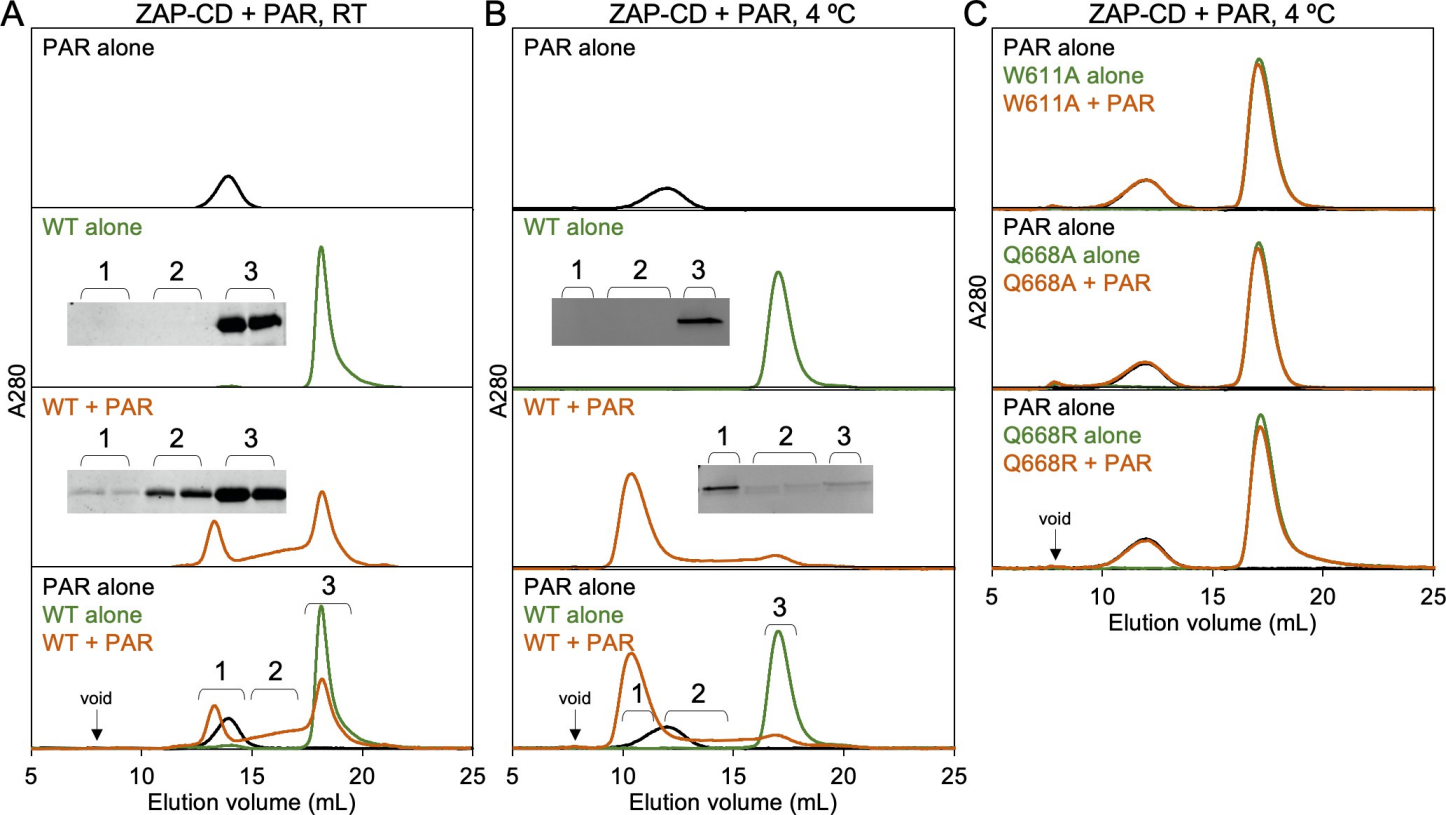
ZAP-CD binds to PAR *in vitro*. (A) Size exclusion binding assay with purified ZAP-CD and PAR, performed at room temperature. The three top panels show individual analytical Superdex 200 size exclusion profiles of purified PAR alone (black), ZAP-CD alone (green) and mixed ZAP-CD and PAR after 20 min incubation (orange). The bottom panel shows an overlay of all three curves. Insets show SDS-PAGE analysis of fractions indicated in the bottom panel. Results are representative of two independent experiments, each done in two replicates. (B) Size exclusion assay performed at 4°C. Results are representative of two independent experiments. (C) Representative results of assays (4°C) performed with the indicated ZAP-CD mutants. Results are representative of two independent experiments.

To directly test whether ZAP interacts with PAR, we enzymatically synthesized and purified PAR polymers *in vitro* [24,25] (S3A Fig). We then used analytical size exclusion chromatography to test for a binding interaction (Fig 5). In this experiment, a positive interaction can be generally expected to manifest in one of two ways. High-affinity binding can result in formation of a stable complex that elutes with an apparent size (strictly speaking, hydrodynamic radius) greater than the early-eluting component. Less stable complexes can dissociate and exchange with the unbound components during the chromatography run to generate an elution profile in which the late-eluting component peak is smeared towards earlier volumes. For these experiments, we used a PAR fraction whose elution volume (S3B Fig) allowed us to distinguish the unbound PAR from both the bound complexes and unbound ZAP-CD. Note that the PAR polymers in this fraction run as an apparent single band on agarose gel electrophoresis (S3C Fig), but are heterogeneous in length and may even constitute both linear and branched forms.

In control experiments, ZAP-CD alone eluted as a single peak from an analytical Superdex 200 column with an elution volume of ~18 mL (Fig 5A, green curve). PAR eluted at an earlier volume of ~14.5 mL from the same column (Fig 5A, black curve). When the two components were mixed prior to sample injection, both types of elution behavior described above were observed. The profile consisted of a peak with an elution volume of ~13.5 mL, which is earlier than either PAR or ZAP-CD alone and indicative of a stable complex; in addition, the trailing ZAP-CD peak was also smeared towards earlier elution volumes (Fig 5A, orange curve). SDS-PAGE confirmed the presence of protein in all the relevant fractions (insets in Fig 5A). These results show that ZAP-CD indeed binds PAR *in vitro*, and that furthermore, two types of ZAP-CD/PAR interactions can be discerned from the exchange behavior of the complexes during size exclusion chromatography.

The above experiments were performed at room temperature, and so we repeated them at 4°C to test if lower temperature would promote more stable binding. Most of the protein shifted to the early-eluting peak when mixed with PAR prior to injection (Fig 5B). This result indicates that the lower temperature indeed disfavored dissociation of the complexes during the chromatography run.

As described above, the putative PAR-binding pocket in ZAP-CD contains a buried glutamine residue, Q668, surrounded by hydrophobic sidechains including W611 (Fig 3D). To confirm the importance of this pocket for PAR binding, we purified and tested ZAP-CD proteins harboring W611A, Q668A or Q668R mutations (Fig 1B). Chromatography was performed at 4°C; more efficient binding was observed with the wild type (WT) protein at this temperature and hence is a more stringent test for loss of binding. The mutant proteins did not bind PAR as evidenced by the elution profiles of the mixed samples, which were simple sums of the profiles of the individual components (Fig 5C). These results confirm that the shifts in elution volume arise from specific interactions between ZAP-CD and PAR, and that these interactions involve binding of an ADP-containing unit of PAR to the WWE pocket.

### ZAP-CD binds PAR in cells

**Fig 6:**
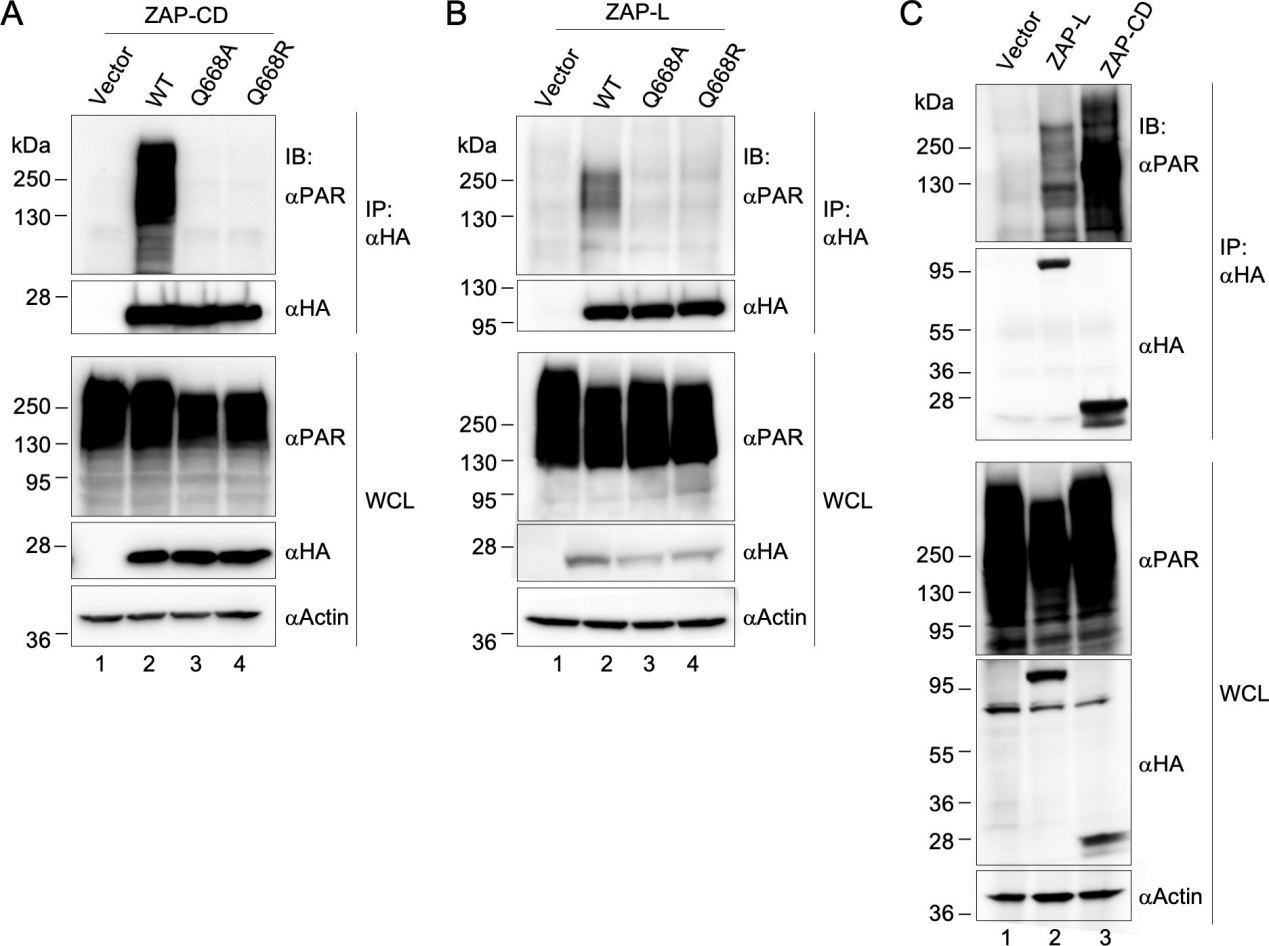
PAR co-immunoprecipitates with ZAP. (A-B) HEK 293T cells were transfected with empty vector or the indicated HA-tagged ZAP-CD constructs (A) or ZAP-L constructs (B). Forty-eight hours later, whole cell lysates (WCL) were subjected to pull-down with anti-HA antibody followed by immunoblotting with the indicated antibodies. Actin was used as loading control. Results are representative of three (ZAP-CD) or two (ZAP-L) independent experiments. (C) Comparison of ZAP-CD and ZAP-L.

To confirm that ZAP interacts with PAR in cells, we overexpressed HA-tagged ZAP proteins in HEK 293T cells and performed co-immunoprecipitation experiments (Fig 6). HA-tagged ZAP-CD efficiently co-precipitated PAR from clarified cell lysates (Fig 6A, lane 2). In contrast, ZAP-CD proteins harboring the Q668A or Q668R mutations did not co-precipitate PAR (Fig 6A, lanes 3 and 4). Experiments performed with HA-tagged ZAP-L, a naturally occurring full-length isoform of ZAP, likewise revealed that ZAP-L could co-precipitate PAR (Fig 6B, lane 2) and that the Q668A and Q668R ZAP-L mutants are impaired (Fig 6B, lanes 3 and 4). These results indicate that the ZAP central domain indeed binds to PAR in cells as predicted by our structural and biochemical analyses. Interestingly, it appeared that substantially more PAR co-immunoprecipitated with ZAP-CD than ZAP-L, taking into account the differences in protein amounts (compare lane 2 in Fig 6A with lane 2 in Fig 6B). We confirmed this with a side-by-side comparison of ZAP-L and ZAP-CD (Fig 6C). Thus, it appears full-length ZAP-L does not bind PAR as efficiently as ZAP-CD.

**Fig 7:**
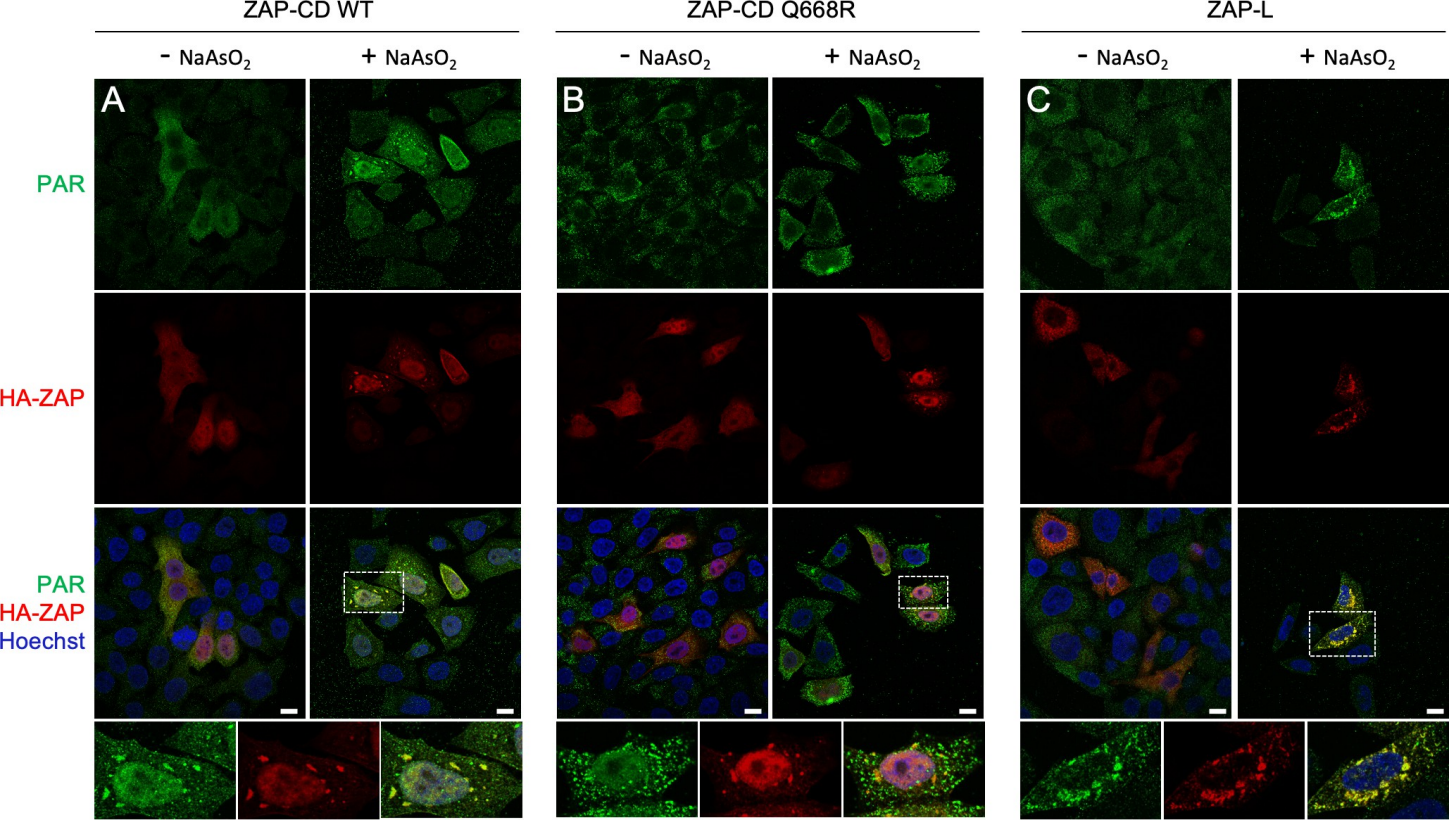
ZAP redistributes with PAR in cytoplasmic puncta. HeLa cells were transiently transfected with (A) vector encoding HA-tagged ZAP-CD, (B) ZAP-CD Q668R mutant, or (C) ZAP-L. Twenty-four hours later, cells were treated with sodium arsenite or mock-treated, fixed, immunostained with anti-HA and anti-PAR primary antibodies followed by dye-conjugated secondary antibodies, and imaged by using fluorescence microscopy. Results are representative of three (ZAP-CD) or two (ZAP-L) independent experiments. Scale bars, 10 μm.

PAR is ubiquitously found in cells but becomes enriched within non-membranous sub-cellular compartments called RNA stress granules upon stress induction or virus challenge. For example, treatment of cells with arsenite (which causes oxidative stress) induces PAR accumulation in stress granules [14]. We took advantage of this property to develop a biochemical assay that directly examines ZAP and PAR association in the cellular setting. HA-tagged ZAP-CD was expressed in HeLa cells under conditions that allowed facile visualization of both ZAP-CD and PAR by immunofluorescence microscopy (Fig 7). As expected, arsenite treatment redistributed PAR into large punctate accumulations in the cytoplasm (Fig 7A–7C, top panels). Control experiments confirmed that these puncta also contained an established stress granule marker (S4A Fig) and that the arsenite-induced PAR accumulations are independent of ZAP (vector only control, S4B Fig). Correspondingly, arsenite treatment also induced the redistribution of ZAP-CD into large puncta, and importantly these ZAP-CD accumulations are visibly co-localized with the PAR accumulations (Fig 7A; quantified in S4C Fig). In contrast, the ZAP-CD Q668R mutant did not significantly re-localize with PAR (Figs 7B and S4C). These results support the conclusion that the ZAP central domain binds PAR, both *in vitro* and in cells. We also performed the redistribution experiment with ZAP-L and obtained similar results (Figs 7C and S4C).

### PAR-binding by the central domain potentiates ZAP antiviral activity

**Fig 8:**
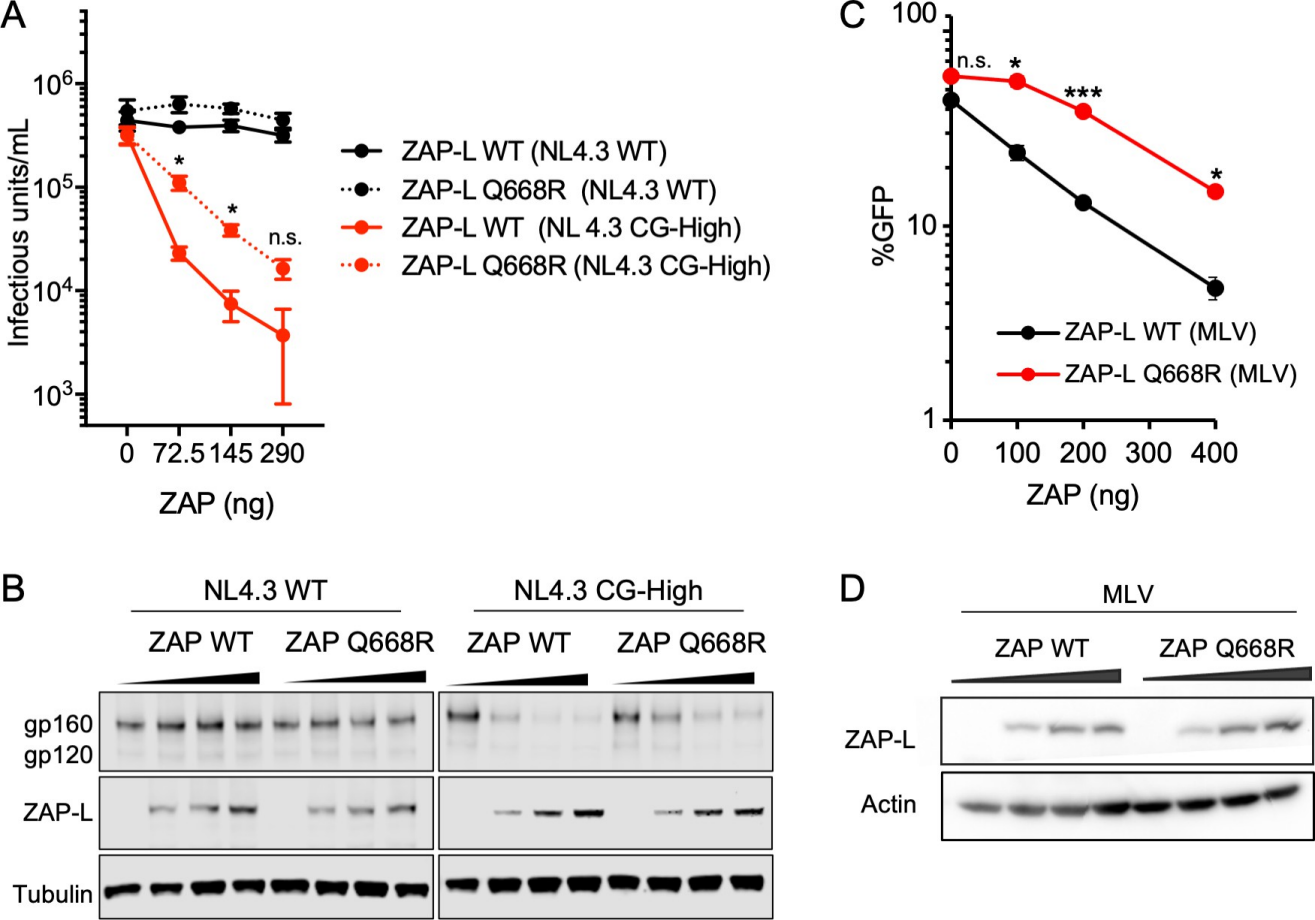
Antiviral potency of ZAP-L is reduced by the Q668R mutation. (A) HEK 293T *ZAP*^-/-^ *TRIM25*^-/-^ cells were transfected with a provirus of either HIV-1 (NL4.3 WT) control or a CpG-enriched mutant (NL4.3 CG-High), together with a plasmid encoding TRIM25 and increasing concentrations of a plasmid encoding WT ZAP-L or the ZAP-L Q668R mutant. After 48 hours, produced virus was harvested, filtered and titered. (B) Immunoblots of whole cell lysates showing expression levels of HIV-1 proteins (gp160 and gp120) and ZAP-L. Tubulin was used as loading control. Results are representative of two independent experiments. (C) The same experiment as in A was performed with MLV. (D) Immunoblots of whole cell lysates showing ZAP-L expression levels from plasmids co-transfected with MLV. Actin was used as loading control. *, p<0.05; ***, p<0.001; ns, not significant.

Having established the biochemical properties of the ZAP central domain both *in vitro* and in cells, we next tested whether its PAR binding activity would affect ZAP’s antiviral function against CpG-enriched HIV-1. We used an engineered HIV-1 mutant, termed (NL4.3 CG-High), that was previously generated by synonymous mutagenesis and contains a higher number of CpGs compared to WT HIV-1 [7]. ZAP directly binds to and directs the degradation of the CpG-rich viral RNA transcripts, thereby reducing viral protein synthesis and infectious virus yield of (NL4.3 CG-High). We transfected ZAP-deficient HEK 293T cells with proviral plasmids encoding WT HIV-1 control (NL4.3 WT) or CpG-enriched virus (NL4.3 CG-High), together with varying amounts of expression vectors encoding either WT ZAP-L or the ZAP-L Q668R mutant. Virus yields were measured 48 h after transfection. While both the WT and CpG-enriched viruses gave similar yields in the absence of ZAP-L, progressively increasing the expression of ZAP-L resulted in corresponding reduction in the yield of infectious units of HIV-1 (NL4.3 CG-High) but not (NL4.3 WT) (Fig 8A). Notably, the PAR-binding deficient ZAP-L mutant (Q668R) exhibited 5- to 10-fold reduced antiviral potency when compared to WT ZAP-L. The diminished antiviral activity was also reflected in the amount of Env protein synthesized in transfected cells (gp160 and gp120, Fig 8B).

Finally, we tested effects on murine leukemia virus (MLV), which is naturally susceptible to the antiviral activity of ZAP (Fig 8C and 8D) [1]. ZAP-deficient HEK 293T cells were transfected with a proviral vector for MLV, together with varying amounts of expression vector for either WT or Q668R ZAP-L and virus yields were quantified as above. Similar to the HIV-1 (NL4.3 CG-High), WT ZAP-L had antiviral activity against MLV while the Q668R ZAP-L mutant exhibited ~3-fold reduced potency.

Taken together, our results confirm that the PAR-binding property of the central domain is not strictly required for ZAP-L-mediated inhibition of virus replication. Nevertheless, loss of the ability to bind PAR correlates with an appreciable decrease in antiviral activity.

## Discussion

While it is established that the N-terminal RNA-binding domain of ZAP containing its first four zinc fingers is both necessary and sufficient to recognize CpG-rich viral RNA and direct their degradation, how the downstream domains contribute to ZAP’s antiviral function remains to be elucidated. Our structural and biochemical studies here reveal that the central regions of ZAP, comprising the fifth zinc finger and two WWE modules, integrate to form a single folded domain. This ZAP central domain displays an electropositive surface and features a prominent pocket in the second WWE module. Our data indicate that this ZAP pocket is analogous the isoADPr-binding sites found in other WWE domains. However, the ZAP pocket does not fully recapitulate all the structural features of the prototype isoADPr pocket in RNF146, suggesting that there may be differences in binding mode. Specifically, we find that the ZAP pocket is configured to accommodate an adenosine diphosphate unit, which is an internal repeating unit of PAR. A single ADP unit without the additional phosphoribose moiety of isoADPr is also found at the free termini of a PAR polymer or in proteins conjugated to a single (mono) ADP-ribose unit (MARylation). Further studies are needed to determine if the ZAP pocket has preference for terminal or internal ADP units of PAR. Moreover, since the composite central domain fold of ZAP has an extended electropositive cleft, we speculate that its ADP pocket may be part of a more extensive binding site for a PAR polymer. Overall, our study provides further support for the idea that WWE domains have a common function as PAR-binding modules [11,24].

How might PAR binding relate to ZAP’s antiviral function? Being an extended polynucleotide, PAR chains have the requisite size and architecture to function as a polyvalent scaffold that facilitates clustering of binding partners [13,26]. Multiple CpGs are required to target an RNA strand for degradation and each ZAP RBD can only bind a single CpG dinucleotide, implying that selective recognition requires formation of a multivalent ZAP/RNA complex [5–7]. Thus, a simple model is that PAR binding by ZAP can facilitate recognition and subsequent RNA processing by promoting local clustering of the protein molecules and thereby shifting the interaction equilibria to favor association. It is possible that such an affinity amplification mechanism may become more acutely important in certain contexts, for example when pathway components are limiting or when viral RNA levels are low.

PAR binding may also regulate ZAP’s sub-cellular distribution. PAR is critical for the formation and maintenance of RNA stress granules [14], and ZAP has been shown to localize to stress granules upon viral infection [18]. ZAP was also identified as a component of granules induced by stress [14,27]. The ZAP RNA-binding domain can independently associate with stress granules [18], although it is not clear whether this occurs through direct PAR binding independent of the central domain or indirectly through bound RNA. Both ZAP-L and ZAP-S are also PARylated, at least under stress conditions [14]. Similarly, at least one ZAP co-factor, TRIM25, has been reported to be associated with stress granules [27–29]. Although it remains to be established whether stress granules are the actual site of antiviral activity, it was recently reported that differential access of the long and short ZAP isoforms to target RNA populations is regulated by sub-cellular localization [30]. Specifically, ZAP-L is targeted to intracellular compartments by a C-terminal posttranslational modification (prenylation [31]) where it can access viral RNA, whereas ZAP-S lacks this targeting signal and remains cytosolic where it accesses a different pool of cellular RNA [30]. Both forms of ZAP contain the central domain, and thus, binding of the central domain to PAR may be an additional mechanism to regulate where and when ZAP engages its targets.

## Materials and methods

### Plasmids

ZAP-L was obtained from Addgene (plasmid #45907). ZAP-CD was generated by using primers containing a Kozak sequence and an N-terminal HA tag, and inserted into pCDNA3-MCS between the EcoRI and NotI sites. ZAP mutants (W611A, Q668A, and Q668R) were generated by using the QuikChange Lightning site-directed mutagenesis kit (Agilent Technologies). *E*. *coli* expression plasmids were generated by sub-cloning from the Addgene plasmid using Gibson assembly. All coding sequences were confirmed by DNA sequencing.

### ZAP-CD purification, crystallization and structure determination

ZAP-CD was expressed with a His_6_-SUMO leader sequence in *E*. *coli* BL21(DE3) cells by using the autoinduction method [32]. The His-tagged fusion protein was purified by using Ni-NTA chromatography, the tag was removed with Ulp1 protease, and the untagged ZAP-CD protein was purified to homogeneity using anion exchange chromatography. The protein was exchanged into storage buffer (20 mM Tris, pH 8, 100 mM NaCl, 1 mM TCEP) by using preparative size exclusion or dialysis.

Crystallization was performed in sitting drops, by mixing protein and precipitant (0.8–1.1 M sodium nitrate, 0.1 M sodium acetate, pH 4.8–6.0) at a volume ratio of 3:1. Crystals were cryo-protected in 20% PEG 400 and diffraction data were collected at the Advanced Photon Source beamline 22-ID. The structure was solved by single anomalous diffraction methods from a selenomethionine dataset, and the data quality was sufficiently high to permit automatic model building by PHENIX software [33] directly from integrated data with only some manual rebuilding required. At the completion of structure refinement, weak but clear difference density was observed in the second WWE domain, suggesting that some small molecule had co-purified and co-crystallized with ZAP-CD. Indeed, the A_260_/A_280_ ratios of purified samples were ~0.6–0.7, indicating the presence of low levels of some A_260_-absorbing component. However, only a small fraction of the protein was bound, because the co-purifying small molecule was not detected by mass spectrometry analysis of the sample used for crystallization. It appears that this bound fraction was the one that crystallized, because the crystals were very sparse and small.

To obtain the ZAP-CD structure in complex with ADPr, freshly purified protein was mixed with excess ADPr (~1 mM) prior to crystallization. In the presence of ADPr, the crystals were much more numerous and much larger. These crystals were cryo-protected in 25% glycerol and diffraction data were collected at the Advanced Photon Source beamline 22-BM. The structure was solved by molecular replacement with the “uncomplexed” ZAP-CD structure above, and refined in PHENIX [33].

All structure statistics are summarized in Table 1. The ZAP-CD structure is deposited in the Protein Data Bank (PDB) as 7KZH. The ZAP-CD/ADPr structure is deposited as 7TGQ.

### Differential scanning fluorimetry

Thermal melting profiles were measured by using a Tycho (NanoTemper), following the manufacturer’s instructions.

### Preparation of PAR

PAR was enzymatically prepared as previously described [25].

### Size exclusion binding assay

Size exclusion was performed in 20 mM Tris, pH 8, 100 mM NaCl, 1 mM TCEP. Four A_280_ absorbance units of ZAP-CD (77 μM) was mixed with equal volume of four A_260_ absorbance units of PAR (296 μM in terms of ADP-ribose subunits), incubated for 20 min, and then injected on a Superdex 200 30/100 column (GE Healthcare) and developed at a flow rate of 0.5 mL/min. For control injections, ZAP-CD or PAR was mixed with buffer alone.

### Cells and plasmid transfections

HEK 293T and HeLa cell lines were maintained in DMEM supplemented with 10% FBS (fetal bovine serum), 100 U/mL penicillin, and 100 μg/mL streptomycin. Transfections were performed by using Hilymax (Dojindo Molecular Technologies) according to the manufacturer’s instructions.

### Immunoprecipitation and immunoblotting

At 48 h after transfection, HEK 293T cells were washed twice with PBS (phosphate-buffered saline) and lysed in IP buffer (50 mM Tris, pH 7.5, 150 mM NaCl, 0.2% Triton X-100) containing 1 μM ADP-HPD (Millipore) and cOmplete Mini EDTA-free inhibitor (Roche) for 20 min (with rotation at 4°C). The lysate was clarified by centrifugation at 4°C for 15 min at 18,800 ×*g*. A final concentration of 10 μg/mL cytochalasin B (Sigma) and 25 μM nocodazole (Sigma) were then added to the supernatant. Samples were then mixed with either anti-HA magnetic beads (Thermoscientific) or protein G magnetic beads (Thermoscientific) coupled with anti-pADPr (Abcam) (beads were pre-blocked with 1–2% BSA). After overnight incubation at 4°C, the beads were washed 4 times with IP buffer, re-suspended in SDS loading buffer, boiled for 5 min, electrophoresed on a Novex 4–20% Tris-Glycine mini-gel (Invitrogen), and then transferred onto PVDF membranes. Mouse anti-poly(ADP-ribose) polymer [10H] (1:1000; Abcam), mouse anti-HA [F-7] (1:3000; Santa Cruz), mouse anti-β-Actin [C4] (1:3000; Santa Cruz), and HRP-conjugated goat antibody to mouse (1:10,000; Azure Biosystems) were used for detection.

### Immunostaining and fluorescence microscopy

At 24 h after transfection, HeLa cells were treated with or without 250 μM sodium arsenite for 30 min. HeLa cells on coverslips were washed twice in PBS and fixed for 15 min in 4% paraformaldehyde and then permeabilized with 0.5% Triton X-100. Coverslips were washed three times in PBS, then blocked with 3% BSA in TBST (Tris-buffered saline supplemented with 0.05% Tween 20) for 30 min. Cells were incubated with primary antibodies at room temperature for 1 h, and then with Alexa-Fluor-conjugated secondary antibodies and Hoechst 33342 (ThermoFisher) at room temperature for 30 min. Coverslips were mounted on glass slides with ProLong Gold antifade solutions (Molecular Probes). Mouse monoclonal anti-poly(ADP-ribose) polymer [10H] (1:50; Abcam) and rabbit monoclonal anti-HA [C29F4] (1:500, Cell Signaling) were used as primary antibodies. Secondary antibodies were donkey anti-mouse IgG H&L Alexa Fluor 647 (1:500; Abcam) and donkey anti-rabbit IgG H&L Alexa Fluor 488 (1:500; Abcam).

Microscopy was performed by using an LSM 880 confocal laser scanning microscope (Zeiss) with a 63× oil immersion objective (NA 1.4) and Zen imaging software (Zeiss). Images were collected simultaneously using the 405, 488 and 633 nm excitation laser lines.

### Antiviral activity assays

HEK 293T *ZAP*^-/-^ *TRIM25*^-/-^ cells were transfected with proviral plasmids of either WT HIV-1 or a CpG-enriched mutant (NL4-3) [7], together with a plasmid encoding TRIM25 and increasing concentrations of a plasmid encoding wild type ZAP-L or mutant ZAP-L (Q668R). Cells were incubated for 48 h at 37°C. Produced virus was then harvested, filtered and titered on MT4-GFP cells to determine infectious units per mL.

Similarly, B-tropic MLV-based retroviral vector pCIG3-B and packaging vectors (pLXIN-GFP and pMD2.G) [34,35] were transfected into HEK 293T *ZAP*^-/-^ *TRIM25*^-/-^ cells, together with a plasmid encoding TRIM25 and increasing concentrations of plasmid encoding WT or Q668R ZAP-L. Cells were incubated for 48 h at 37°C. Produced virus was then harvested, filtered and used to transduce HeLa cells. Cells expressing GFP were determined by flow cytometry (Guava easyCyte 8HT) and analyzed using GuavaSoft version 2.7.

Statistical significance was assessed using Brown-Forsythe and Welch ANOVA.

## Supporting information

S1 Fig Comparison of Z5 to the Z1-Z4 zinc fingers in the RBD. (A) Structure-based sequence alignment. The zinc-coordinating residues in the CCCH motif are indicated in bold. (B) The Z1, Z2, Z3 and Z4 modules from the non-RNA-bound human ZAP structure (PDB 6UEI [5]) were superimposed on structurally equivalent residues of Z5. (C) Comparison of Z5 from human ZAP with the Z2 modules from RNA-bound human ZAP (PDB 6UEJ [5]) and RNA-bound mouse ZAP (PDB 6L1W [6]). Tyrosines in Z2 that contact the RNA CpG motif are not conserved in Z5.
(TIFF)

S2 Fig Identification of a bound molecule in ZAP-CD crystallized without added ligand. (A) Electron density (mesh) after refinement. The 2mFo-DFc map is shown in two contours, 1σ in dark blue and 2σ in light blue. Residual mFo-DFc density at 3σ is in green. (B) The structure of the RNF146 WWE domain (gray) with bound isoADPr (magenta) (PDB 3V3L [11]) was superimposed on the ZAP-CD WWE2 module (orange), shown in the same orientation as in A. The residual unbiased mFo-DFc density (green mesh, 3σ) matches the position of the bound ligand in the RNF146 structure. (C) Close-up view of the superimposed structures, showing only the residual densities from ZAP (mFo-DFc, 3σ, green and 2mFo-DFc, 1σ, blue) and the RNF146-bound isoADPr.
(TIFF)

S3 Fig Synthesis and preparation of PAR. (A) Histones were PARylated by incubation with recombinant PARP1 enzyme and NAD^+^ [25]. After proteolytic digestion to remove the proteins, released PAR polymers were purified. (B) Size exclusion profile on a preparative Superdex 200 column, after resuspension of the isopropanol precipitate. (C) Agarose gel electrophoresis profiles of fractions from the chromatography run in B.
(TIFF)

S4 Fig PAR redistribution upon stress induction with arsenite. (A-B) HeLa cells were transiently transfected with empty vector. Twenty-four hours later, cells were treated with sodium arsenite or mock-treated, fixed, immunostained with the indicated primary antibodies followed by dye-conjugated secondary antibodies, and imaged by using fluorescence microscopy. Results are representative of two independent experiments. Scale bars, 10 μm. (C) Quantification of ZAP redistribution with PAR in arsenite-treated cells. At least 10 fields were randomly selected, and the number of PAR puncta that also co-stained for ZAP were counted in the ZAP-expressing cells seen in each field. ****, p<0.0001; ns, not significant.
(TIFF)
