# Supplementary figures and images for: Poly(ADP-ribose) potentiates ZAP antiviral activity
Source: PLoS Pathog. 2022 Feb 7;18(2):e1009202. doi: 10.1371/journal.ppat.1009202 (PMC8853533; doi:10.1371/journal.ppat.1009202)

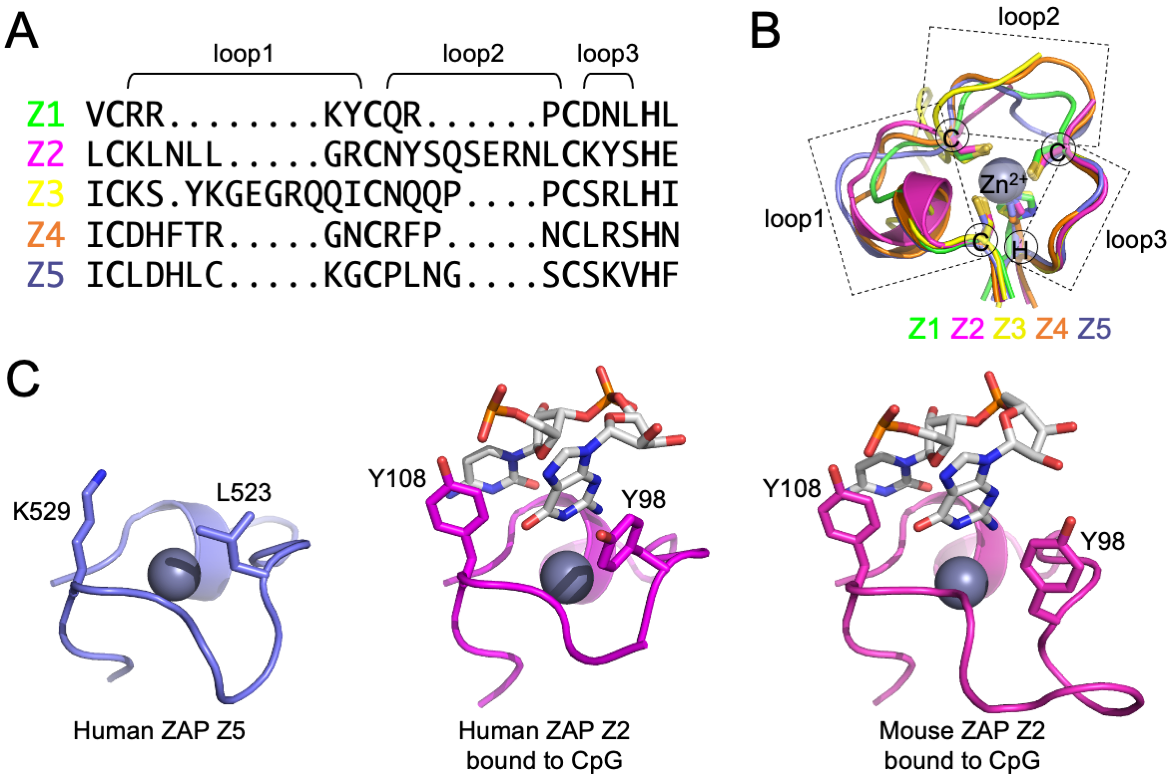

Supplement: S1 Fig — (A) Structure-based sequence alignment. The zinc-coordinating residues in the CCCH motif are indicated in bold. (B) The Z1, Z2, Z3 and Z4 modules from the non-RNA-bound human ZAP structure (PDB 6UEI [5]) were superimposed on structurally equivalent residues of Z5. (C) Comparison of Z5 from human ZAP with the Z2 modules from RNA-bound human ZAP (PDB 6UEJ [5]) and RNA-bound mouse ZAP (PDB 6L1W [6]). Tyrosines in Z2 that contact the RNA CpG motif are not conserved in Z5. (TIFF) [file ppat.1009202.s001.tiff]

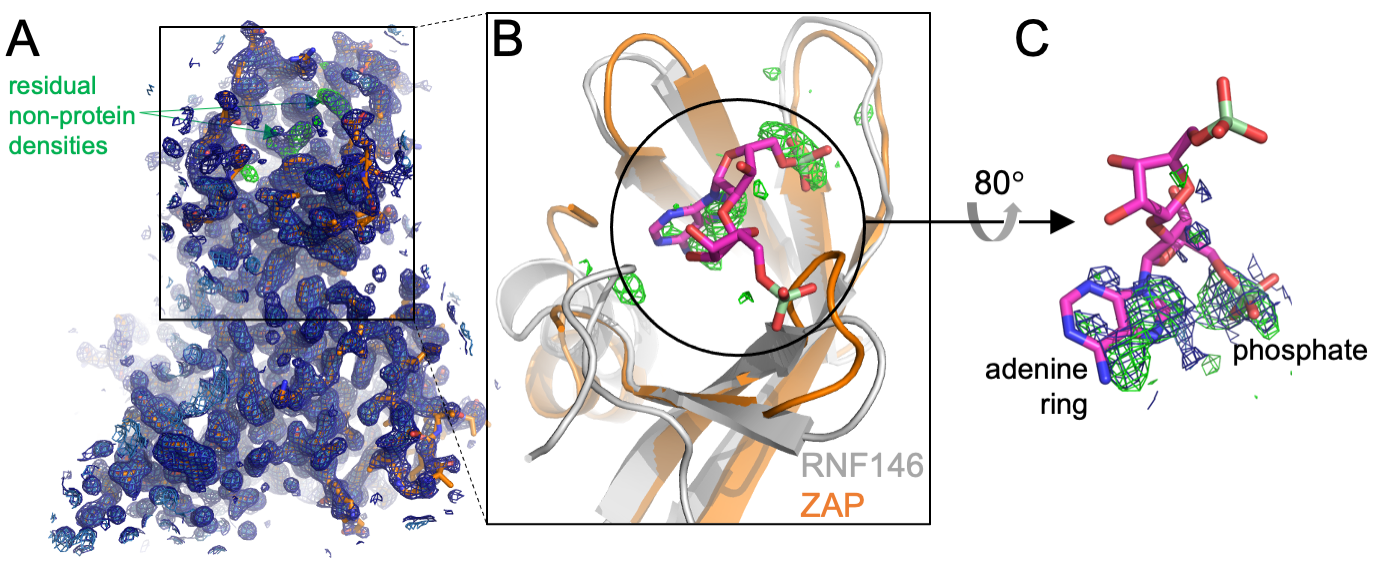

Supplement: S2 Fig — (A) Electron density (mesh) after refinement. The 2mFo-DFc map is shown in two contours, 1σ in dark blue and 2σ in light blue. Residual mFo-DFc density at 3σ is in green. (B) The structure of the RNF146 WWE domain (gray) with bound isoADPr (magenta) (PDB 3V3L [11]) was superimposed on the ZAP-CD WWE2 module (orange), shown in the same orientation as in A. The residual unbiased mFo-DFc density (green mesh, 3σ) matches the position of the bound ligand in the RNF146 structure. (C) Close-up view of the superimposed structures, showing only the residual densities from ZAP (mFo-DFc, 3σ, green and 2mFo-DFc, 1σ, blue) and the RNF146-bound isoADPr. (TIFF) [file ppat.1009202.s002.tiff]

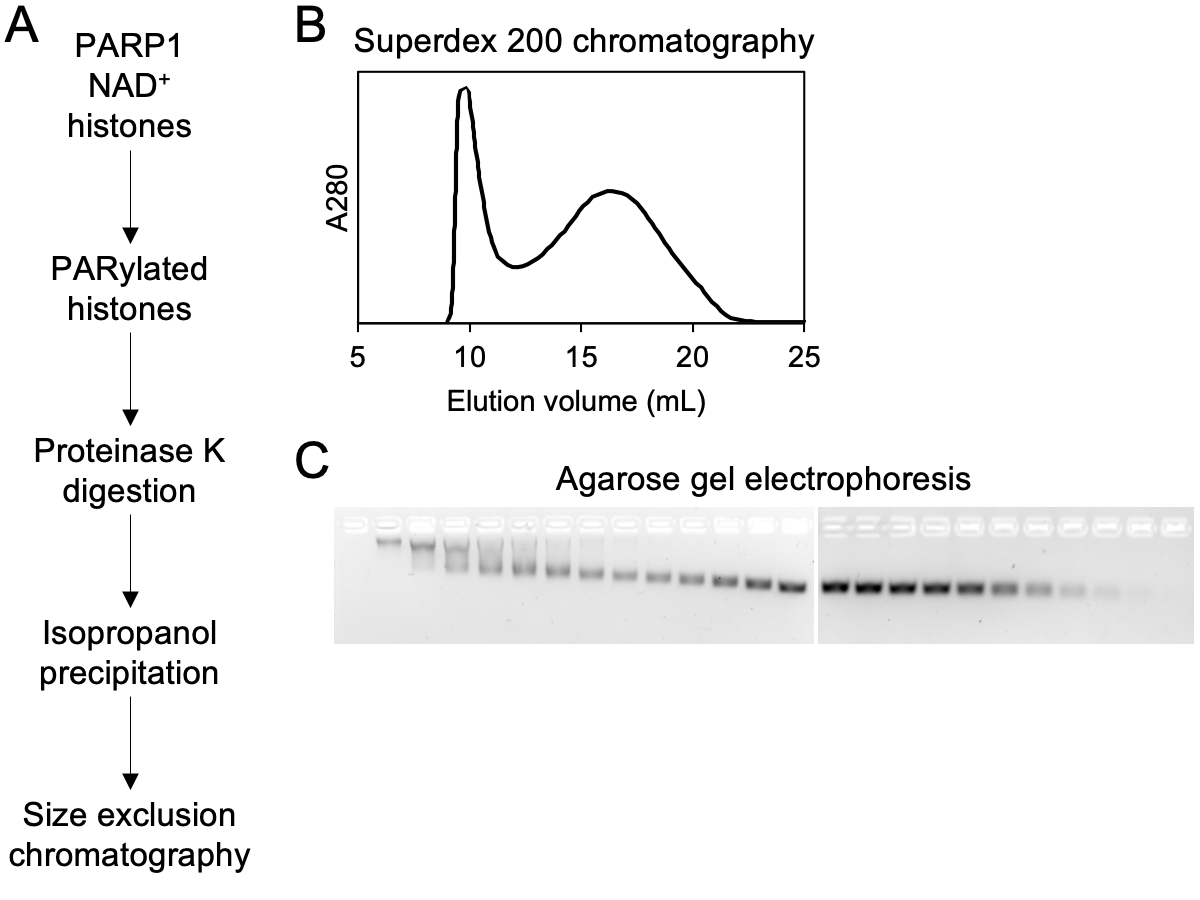

Supplement: S3 Fig — (A) Histones were PARylated by incubation with recombinant PARP1 enzyme and NAD+ [25]. After proteolytic digestion to remove the proteins, released PAR polymers were purified. (B) Size exclusion profile on a preparative Superdex 200 column, after resuspension of the isopropanol precipitate. (C) Agarose gel electrophoresis profiles of fractions from the chromatography run in B. (TIFF) [file ppat.1009202.s003.tiff]

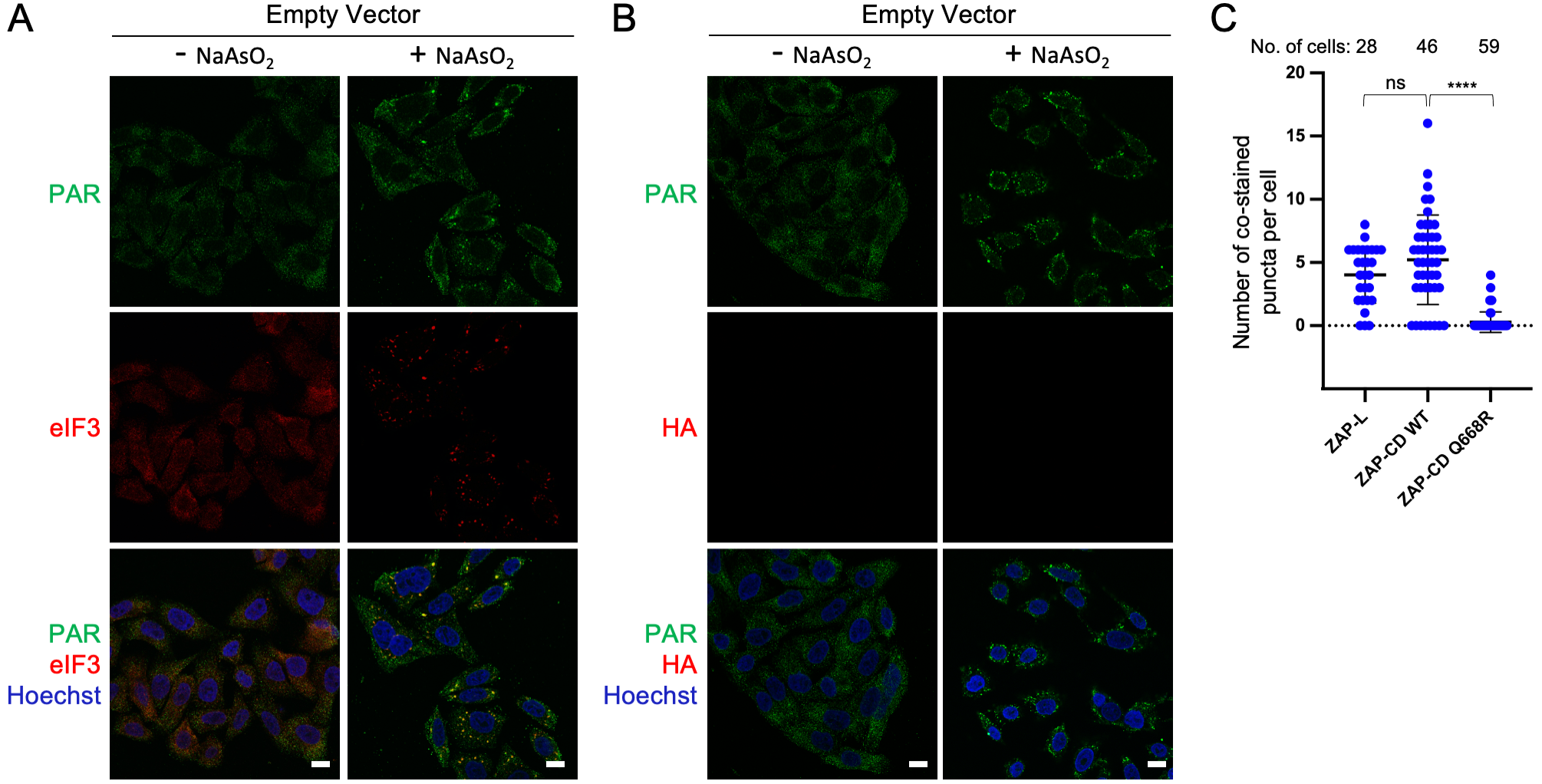

Supplement: S4 Fig — (A-B) HeLa cells were transiently transfected with empty vector. Twenty-four hours later, cells were treated with sodium arsenite or mock-treated, fixed, immunostained with the indicated primary antibodies followed by dye-conjugated secondary antibodies, and imaged by using fluorescence microscopy. Results are representative of two independent experiments. Scale bars, 10 μm. (C) Quantification of ZAP redistribution with PAR in arsenite-treated cells. At least 10 fields were randomly selected, and the number of PAR puncta that also co-stained for ZAP were counted in the ZAP-expressing cells seen in each field. ****, p<0.0001; ns, not significant. (TIFF) [file ppat.1009202.s004.tiff]
